# Tenecteplase Versus Reteplase in Acute Myocardial Infarction: A Network Meta-Analysis of Randomized Clinical Trials

**DOI:** 10.22037/ijpr.2019.1100743

**Published:** 2019

**Authors:** Majid Zia-Behbahani, Hossein Niknahad, Javad Kojuri, Mahmood Salesi, Mojtaba jafari, Khosro Keshavarz

**Affiliations:** a *Student Research Committee, Shiraz University of Medical Sciences, Shiraz, Iran. *; b *Department of Pharmacology and Toxicology, Faculty of Pharmacy, Shiraz University of Medical Sciences, Shiraz, Iran. *; c *Quality Improvement in Clinical Teaching Research Center, Shiraz Education Center, Faculty of Medical Education, Shiraz University of Medical Sciences, Shiraz, Iran. *; d *Chemical Injuries Research Center, Systems Biology and Poisonins Institute, Baqiyatallah University of Medical Science, Tehran, Iran.*; e *Health Human Resources Research Center and Department of Health Economic, School of Management and Medical Informatics, Shiraz University of Medical Sciences, Shiraz, Iran.*

**Keywords:** Tenecteplase, Reteplase, Alteplase, Acute myocardial infarction, Network meta-analysis, Clinical efficacy

## Abstract

Acute myocardial infarction (AMI) is the leading cause of death throughout the world. One of the standard approaches to treatment of AMI is fibrinolysis. The study was conducted to evaluate the clinical efficacy of tenecteplase versus reteplase through network meta-analysis for AMI. Randomized trials were comprehensively searched in PubMed, Scopus, Cochrane library, and Web of Science using appropriate strategies. Quality assessment was done for the papers. The primary and secondary end-points were mortality, TIMI grade 3 flow at 90 min, death or non-fatal stroke, infarction, total stroke and major bleeding. Odds ratios (OR) were computed (95% confidence intervals). After screening 27325 records, eight articles were included with total patients of 49875 to the meta-analysis. Indirect comparison of tenecteplase vs. reteplase showed no significant differences in the risk of mortality (OR = 0.98, *p > 0.05*), TIMI grade 3 flow at 90 min (OR = 0.77, *p > 0.05*), death or non-fatal stroke (OR = 1.04, *p > 0.05*), infarction (OR = 1.11, *p > 0.05*), total stroke (OR = 2.71, *p *> *0.05*), and major bleeding (OR = 0.81, *p > 0.05*) (all *p > 0.05*). Indirect comparison suggests similar efficacy and safety of tenecteplase and reteplase. Hence, the use of each one of the two medicines depends on price, facility, and accessibility of the medicine.

## Introduction

According to World health organization (WHO) and centers for disease control and prevention (CDC) reports, ischemic heart disease is the leading cause of death in Iran and the United States in 2016 and is one of the major killers in the world (1). One of its main manifestations is acute myocardial infarction (AMI), which in most cases is the outcome of a thrombus or clot forming on top of a ruptured atherosclerotic plaque, resulting in the obstruction of the blood flow through the coronary artery with or without concomitant vasoconstriction (2, 3). Myocardial infarction caused by complete coronary artery occlusion begins to develop after 15–30 min of severe ischemia and progresses from the sub-endocardium to the sub-epicardium (4). After ischemia and lack of oxygen, death of cardiac myocytes occurs. Acute myocardial infarction can be defined from a number of different perspectives related to clinical, electrocardiographic (ECG), biochemical, and pathologic characteristics (4, 5). Two definitions for acute myocardial infarction have been established as ST-segment Elevation MI and Non ST-segment Elevation MI. 

AMI is managed through two approaches: percutaneous coronary interventions (PCI) and fibrinolytic means. Although primary PCI has shown better clinical outcome than fibrinolytic therapy, e.g. more effective restoration of patency, less re-occlusion, improved residual left ventricular function (4, 6-12), other studies have reported a progressive reduction of mortality (13) and showed that the patients treated within the first 2h had more reduction of mortality than those treated later (44% vs 20%) (4, 14).

On average, one third of all cases of myocardial infarction lead to death before hospitalization, most of them occurring within the first hour after the onset of acute symptoms (15-17). Also, survival rates improve after a heart attack if treatment begins within 1 h (18). 

Fibrinolytic medicines used in AMI include streptokinase, anistreplase, alteplase, reteplase, and tenecteplase. Streptokinase, anistreplase is used less than others due to hypotension and allergic reaction. Alteplase belonging to the first recombinant generation of thrombolytics is identical to native plasminogen activator (t-PA) (19) and produced by recombinant DNA technology (20). It is used in acute ischemic stroke and pulmonary embolism besides AMI (21). The route of its administration is front-loaded. 

Reteplase is a second-generation recombinant plasminogen activator and may cause a higher frequency of bleeding than alteplase due to more fibrinogen depletion (19). It is only used in AMI (21) and administered through two IV bolus injections 30 min apart (3).

Tenecteplase is a bioengineered variant of t-PA which has full fibrinolytic activity (20, 22). The features include reduced drug clearance (4 times more slowly from plasma than native tPA, increased bioavailability, increased area under curve (AUC) (19). It is only used in AMI (21) and is currently under investigation to be used for acute ischemic stroke. The route of administration is a single IV bolus injection (3).

**Figure 1:**
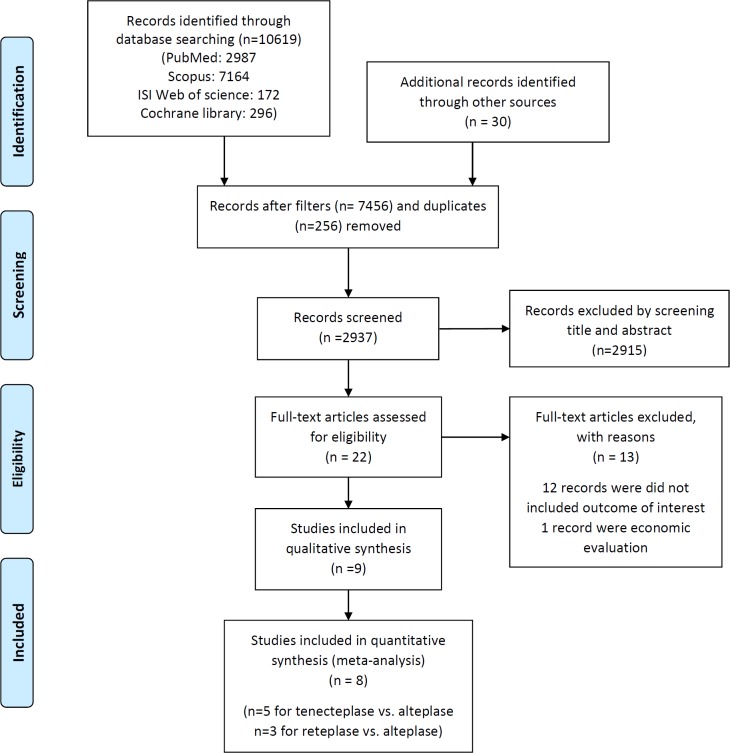
Flow-Chart Identifying Eligible Studies

**Figure 2:**
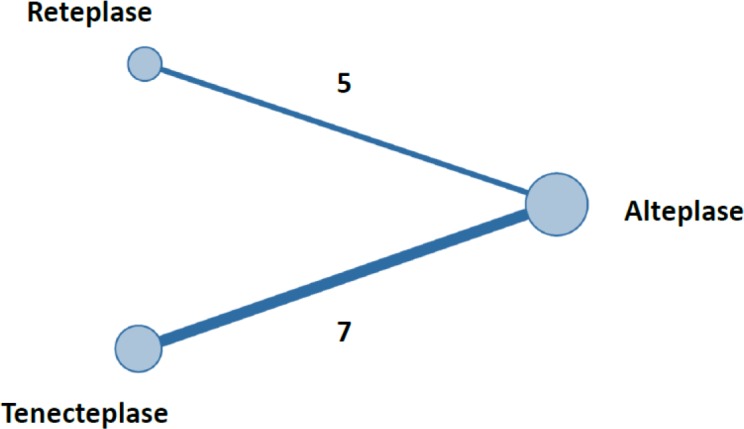
Network plot between groups

**Table1:** Summarized characteristics of the included studies

**Study,**	**Study**	**No. of patients**	**No. of patients**		**Gender , female (number)**		**Intervention**		**Reported Endpoints**
**Year**	**design**		**Group1**	**Group2**	**Group1**	**Group2**	**Group1**	**Group2**	
**Anonymous, 1999**	Prospective	16949	8488	8461	1944	1971	Alteplase (≤100mg)	Tenecteplase (30–50 mg)	mortality, death or non-fatal stroke, infarction, total stroke, major bleeding
**Topol et al., 1997**	Prospective	15059	4921	10138	1338	2788	Alteplase (≤100mg)	Reteplase (10 MU double blous)	mortality, death or non-fatal stroke, infarction, total stroke, major bleeding
**Smalling et al., 1995**	Prospective	300	154	146	42	26	Alteplase (≤100mg)	Reteplase (15MU)	mortality, TIMI grade 3 flow at 90 min, death or non-fatal stroke, infarction, total stroke, major bleeding
**Bode et al., 1996**	Prospective	324	155	169	29	41	Alteplase (≤100mg)	Reteplase (10 MU double blous)	mortality, TIMI grade 3 flow at 90 min, death or non-fatal stroke, infarction, total stroke, major bleeding
**Cannon et al., 1998**	Prospective	613	311	302	67	69	Alteplase (≤100mg)	Tenecteplase (30mg)	mortality, TIMI grade 3 flow at 90 min, death or non-fatal stroke, infarction, total stroke, major bleeding
**Liang et al., 2007**	Prospective	110	52	58	10	10	Alteplase (≤100mg)	Tenecteplase (30–50 mg)	mortality, TIMI grade 3 flow at 90 min, total stroke, major bleeding
**Binbrek et al., 2004**	Prospective	266	134	132	NR	NR	Alteplase (≤100mg)	Tenecteplase (30–50 mg)	mortality, infarction, total stroke, major bleeding
**Smalling et al., 1995**	Prospective	306	154	152	42	35	Alteplase (≤100mg)	Reteplase (10 + 5MU)	mortality, TIMI grade 3 flow at 90 min, death or non-fatal stroke, infarction, total stroke, major bleeding
**Smalling et al., 1995**	Prospective	308	154	154	42	35	Alteplase (≤100mg)	Reteplase (10 + 10MU)	mortality, TIMI grade 3 flow at 90 min, death or non-fatal stroke, infarction, total stroke, major bleeding
**Cannon et al., 1998**	Prospective	459	311	148	67	45	Alteplase (≤100mg)	Tenecteplase (40mg)	mortality, TIMI grade 3 flow at 90 min, death or non-fatal stroke, infarction, total stroke, major bleeding
**Cannon et al., 1998**	Prospective	387	311	76	67	20	Alteplase (≤100mg)	Tenecteplase (50mg)	mortality, TIMI grade 3 flow at 90 min, death or non-fatal stroke, infarction, total stroke, major bleeding
**Sinnaeve et al., 2003**	Prospective	15724	7885	7839	1892	1803	Alteplase (≤100mg)	Tenecteplase (30–50 mg)	mortality

Note: Some trials evaluated different dosages of one medicine; then the data of each dosage form were listed separately in the Table.

**Table 2:** Quality assessment of RCTs through jadad scale

**Study**	**Randomization**	**Double-blind**	**Withdrawals**	**Total Score**
(Van de Werf, 1999) (26)	2	2	1	**5**
(Binbrek *et al *., 2004) (27)	2	0	1	**3**
(Bode *et al*., 1996) (28)	2	0	1	**3**
(Cannon et al., 1998) (29)	2	0	1	**3**
(Liang *et al*., 2007) (30)	2	0	1	**3**
(Smalling *et al*., 1995) (31)	2	1	1	**4**
(Topol *et al*., 1997) (32)	2	0	1	**3**
(Sinnaeve *et al*., 2003) (33)	2	2	1	**5**

**Table 3:** Direct comparison of tenecteplase versus alteplase and reteplase versus alteplase. Values are expressed as odds ratio (95% confidence interval)

**Endpoints**	**Drug1**	**Drug2**	**Frequency**	**Heterogeneity test** **(I** **2** **%, Chi2) [P-value]**	**Pooled OR (CI 95%)**	***p*** **-value**
Mortality1	Alteplase	Tenecteplase	7	(0%,2.70) [0.84]	0.99 (0.92,1.07)	0.88
	Alteplase	Reteplase	5	(21.4%,5.09) [0.28]	0.97(0.86,1.11)	0.72
TIMI grade 3 flow at 90 min2	Alteplase	Tenecteplase	4	(20%,3.75) [0.29]	1.14 (0.91,1.41)	0.25
	Alteplase	Reteplase	4	(70.5%,10.18) [0.02]	0.88(0.57,1.35)	0.57
Death or non-fatal stroke1	Alteplase	Tenecteplase	4	(0%,1.62) [0.65]	0.99(0.89,1.12)	0.98
	Alteplase	Reteplase	5	(29.4%,5.66) [0.23]	1.03(0.91,1.17)	0.64
Infarction2	Alteplase	Tenecteplase	5	(15.6%,4.74) [0.31]	0.91(0.71,1.08)	0.28
	Alteplase	Reteplase	5	(53.5%,8.61) [0.07]	1.01(0.52,1.99)	0.96
Total stroke2	Alteplase	Tenecteplase	6	(0%,2.65) [0.75]	0.92(0.74,1.15)	0.47
	Alteplase	Reteplase	5	(51.1%,8.18) [0.08]	2.49(0.88,7.04)	0.08
Major bleeding1	Alteplase	Tenecteplase	5	(42.6%,6.97) [0.14]	1.32(1.16,1.50)	0.00
	Alteplase	Reteplase	5	(0%,3.38) [0.49]	1.07(0.95,1.21)	0.26

Meta-analysis (Fixed effect).

Meta-analysis (Fixed & Random effect).

**Table 4:** Indirect comparison between tenecteplase and reteplase through the common comparator, alteplase. Values are expressed as odds ratio

**Endpoints**	**Drug1**	**Drug2**	**OR (SE(In(OR))**	***p*** **-value**
Mortality	Tenecteplase	Reteplase	0.98(0.1)	*p>0.05*
TIMI grade 3 flow at 90 min	Tenecteplase	Reteplase	0.77(0.47)	*p>0.05*
Death or non-fatal stroke	Tenecteplase	Reteplase	1.04(0.12)	*p>0.05*
Infarction	Tenecteplase	Reteplase	1.11(0.41)	*p>0.05*
Total stroke	Tenecteplase	Reteplase	2.71(0.64)	*p>0.05*
Major bleeding	Tenecteplase	Reteplase	0.81(0.13)	*p>0.05*

This study was conducted to investigate clinical effectiveness of tenecteplase versus reteplase for patients suffering AMI. Unfortunately, no direct comparison is available on tenecteplase vs. reteplase. Hence, indirect comparison of meta-analysis was performed with regard to alteplase as a common comparator. 

*Method*

*Data resources and search strategy*

Electronic databases including PubMed, Scopus, Cochrane library, and Web of Science were comprehensively searched using appropriate strategies, for randomized trials comparing alteplase, tenecteplase, and/or reteplase in patients with AMI until December 31, 2016 . Keywords used included acute myocardial infarction, tenecteplase, alteplase, reteplase, pharmacology; pharmacotherapy, medication therapy, and drug therapy (see Appendix 1).

*Inclusion and exclusion criteria*

Inclusion criteria were randomized clinical trials (RCTs) comparing tenecteplase vs. alteplase and reteplase vs. alteplase with English language restriction and follow-up of at least 1 month.

Exclusion criteria included animal studies, studies without control group, observational studies, review studies, and economical studies. In addition, studies not approved by ethics committee and without obtaining informed consent from patients were the criteria for exclusion.

*Quality assessment*

Quality assessment of the trials was undertaken through jadad scale system in which each trial was scored between zero and five, according to randomization, double blinded and withdrawal or dropout (23). Studies which received a Jadad score of between three and five were entered into the network meta-analysis. 

*Primary and secondary endpoints*

Primary endpoints included mortality and TIMI grade 3 flows at 90 min. Secondary endpoints included death or non-fatal stroke, infarction, total stroke, and major bleeding. The endpoints were evaluated in at least two trials.

*Data analysis*

*Meta- analysis*

To perform the meta-analysis, PICO included:

P (population): patients suffering AMI. 

I (intervention): tenecteplase.

C (comparators): reteplase or alteplase.

O (outcomes): mortality, TIMI grade 3 flow at 90 min, death or non-fatal stroke, infarction, total stroke, major bleeding. 

*2.6.2. Statistical Analysis*

There were no randomized controlled trials comparing the effects of tenecteplase with those of reteplase directly. However, tenecteplase could be compared with reteplase indirectly through alteplase for various outcomes.

For various outcomes, the pooled odds ratios from randomized trials in the systematic review of tenecteplase compared with alteplase and reteplase with alteplase were computed using random and fixed effects model meta-analysis. Cochran′s Q test and I2 index, used with *P-*value <0.1 were applied to assess heterogeneity among the RCTs included in meta-analysis. In case of homogeneity, fixed-effects model was used because it assumes the estimated effect sizes only differ due to sampling error but in contrast, rejecting the homogeneity assumption can lead to applying a random-effects model that includes both within and between studies variability. To assess heterogeneity and for calculation of direct & indirect effects, “metan” and “indirect” commands in STATA 11.2 were used.

For calculating indirect effect, Bucher *et al*. method was used (24, 25). In this method, the effects of tenecteplase (TNK) relative to reteplase (rPA) can be estimated indirectly through using the direct estimators for the effects of alteplase (tPA) relative to tenecteplase (effect _TNK,_ _tPA_) and alteplase relative to reteplase (effect _rPA, tPA_):

Effect _TNK,_ _rPA_= effect_ TNK,_ _tPA_ – effect _rPA, tPA_

The indirect estimator variance of Effect _TNK,_ _rPA_ is the sum of the direct estimators’ variances:

Variance_ TNK,_ _rPA_ = variance_ TNK,_ _tPA_ + variance_ rPA, tPA_

## Results

*Study screening, characteristics, and quality of included studies*

After screening 27325 records, eight articles were included with total patients of 49875 to the meta-analysis (Figure 1). Also, the summary of the characteristics of the included studies is shown in Table1.

Quality of the included studies was performed through jadad scale system and the studies received scores between three and five (Table 2). 

*Outcomes*

The plot of network meta-analysis is shown in Figure 2. As seen in Figure 2, the numbers of RCTs data comparing tenecteplase with alteplase and reteplase with alteplase were seven and five, respectively.

Direct comparison of tenecteplase versus alteplase and reteplase versus alteplase is presented in Table 3. Results showed that there were no significant differences between alteplase and each of the two other drugs in terms of mortality and TIMI grade 3 flow at 90 min, death or non-fatal stroke, infarction, and total stroke (*p > 0.05*). Alteplase had a significant difference with tenecteplase in the risk of major bleeding and its rate was 1.3-fold greater than tenecteplase (*p < 0.05*). Despite this significant difference, alteplase and reteplase had no difference (*p > 0.05*). 

Indirect comparison of tenectplase vs. reteplase through the common comparator, alteplase is presented in Table 4; the results showed no significant differences in the risk of mortality (OR = 0.98, *p > 0.05*), TIMI grade 3 flow at 90 min (OR = 0.77, *p > 0.05*), death or non-fatal stroke (OR = 1.04, *p > 0.05*), infarction (OR = 1.11, *p > 0.05*), total stroke (OR = 2.71, *p > 0.05*), major bleeding (OR = 0.81, *p > 0.05*) (all *p > 0.05*). Hence, the two drugs had clinically the same effectiveness and safety.

## Discussion

This study reported for the first time in the literature the indirect comparison of clinical efficacy between tenecteplase versus reteplase in patients suffering AMI. Since there were no studies comparing directly tenecteplase with reteplase, alteplase as common comparator was used. Thus, network meta-analysis was undertaken to evaluate indirectly clinical effectiveness of tenecteplase vs. reteplase. As mentioned in the results, there were no significant differences between tenecteplase vs. reteplase in the risk of mortality, TIMI grade 3 flow at 90 min, death or non-fatal stroke, infarction, total stroke, and major bleeding.

AMI is treated through angioplasty or fibrinolysis approach. Angioplasty is superior to fibrinolysis due to better clinical outcomes; Fibrinolysis showed lower mortality rate in early signs of AMI, especially if treatment begins within 1 h. Moreover, prehospital fibrinolytic therapy may result in high reperfusion outcome since the majority of patients can be treated within 2 h of symptom onset (34).

By contrast, major contra-indication of fibrinolysis approach is bleeding (35). In addition, the rate of bleeding is different among fibrinolysis medicines. For example, tenecteplase had less bleeding than alteplase. Also, intracerebral hemorrhage is the worst complication of fibrinolysis therapy (35). 

Alteplase is a second-generation thrombolytic agent that is administered usually as front-loaded, when tenecteplase and reteplase belong to the third-generation thrombolytic agents, administered as a double bolus and as a single bolus, respectively. Compared with alteplase, tenecteplase and reteplase have greater angiographic patency rate in patients with acute myocardial infarction although it is not significant (36). On the other hand, results of our and another study indicated bleeding risk of alteplase was greater than tenecteplase and also lower than reteplase (19). However, in comparison with alteplase in its front-loaded dose, reteplase and tenecteplase are superior in their application (37). 

ASSENT-2 reported that no differences were found between alteplase and tenecteplase, in the risk of mortality, total stroke, haemorrhagic stroke, or re-infarction, (26). Furthermore, higher major bleeding was observed with alteplase. When in the subgroup analyses of mortality were done within 30–35 days, tenecteplase was better than alteplase in patients treated within 4 h of symptom onset (3, 38). 

Comparison between alteplase and reteplase as to the risk of mortality, total stroke, haemorrhagic stroke, major bleeds or re-infarction made no difference in GUSTO-3(32). Despite this, better mortality benefit in late‐treated patients with alteplase was observed (38).

Some meta-analysis studies have been conducted for comparison of fibrinolysis medicines in AMI. In the year 2003, four medicines, streptokinase, alteplase, reteplase, and tenecteplase were evaluated on clinical efficacy (39). Results showed no significant differences among them in terms of mortality when streptokinase had the lowest rate in incidence of stroke. By contrast, streptokinase caused allergic reactions. Thus, the choice of using these medicines is based on the conditions. 

Also, Dundar *et al*. found out four medicines, i.e. streptokinase, alteplase, reteplase, and tenecteplase, had no significant difference in the risk of mortality (38). 

As there is no direct comparison of tenecteplase and reteplase, indirect comparison was undertaken and results of an indirect comparison and direct comparison may not be equal (40), and cautions must be adopted when results of indirect comparisons are interpreted (41). Systematic review and quality assessment of trials should be undertaken to decrease biases. 

By contrast, the results of direct and indirect comparison were observed in the same quality (42). When the results are significantly different, first validity and generalizability of trials should be checked to find the main causes (41). For example, one meta-analysis study suggested to use magnesium in AMI, when large trials refuted to use it (38). 

Trials included in our analysis were in appropriate quality, selected by acceptable methodology and assessed by jadad scale system. The analysis was conducted with respect to primary and secondary endpoints evaluated in trials. Due to lack of direct comparison of tenecteplase and reteplase, alteplase was applied as the common comparator. 

In conclusion, this meta-analysis study indicated similar efficacy and safety of tenecteplase and reteplase. Hence, the use of each one of two medicines depends on price, facility, and accessibility of the medicine.
