# Supplementary material for: Tenecteplase Versus Reteplase in Acute Myocardial Infarction: A Network Meta-Analysis of Randomized Clinical Trials
Source: Iran J Pharm Res. 2019 Summer;18(3):1622–31. doi: 10.22037/ijpr.2019.1100743 (PMC6934957; doi:10.22037/ijpr.2019.1100743)
Supplement: Supplement [file ijpr-18-1622-s001.pdf]

Supplementary Materials for

Tenecteplase Versus Reteplase in Acute Myocardial Infarction: A Network Meta-Analysis of Randomized Clinical Trials

Majid Zia-Behbahani, Hossein Niknahad, Javad Kojuri, Mahmood Salesi, Mojtaba jafari and Khosro Keshavarz*

To whom correspondence should be addressed: khkeshavarz2007@gmail.com

Volume 18, Issue 3 (Summer 2019)

This PDF file include

Table S1

Table S1. Search strategy for clinical trials in databases.

#1	tenecteplase AND (pharmacology OR pharmacotherapy OR drug Therapy OR medication therapy)
#2	reteplase AND (pharmacology OR pharmacotherapy OR drug Therapy OR medication therapy)
#3	alteplase AND (pharmacology OR pharmacotherapy OR drug Therapy OR medication therapy)
#4	#1 OR #2 OR #3
#5	acute myocardial infarction OR myocardial infarction
#6	#4 AND #5
